# Confrontation of endolymphatic hydrops diagnosis on 3-Tesla MRI to clinical and audiovestibular findings in Meniere's disease

**DOI:** 10.3389/fneur.2023.1105461

**Published:** 2023-01-26

**Authors:** Sarah Diorflar, Caroline Guigou, Edouard Daguet, Jean-Loup Bensimon, Michel Toupet, Alexis Bozorg-Grayeli

**Affiliations:** ^1^Department of Otolaryngology, Dijon University Hospital, Dijon, France; ^2^ImVia, Université Bourgogne Franche-Comté, Dijon, France; ^3^Louis Neel Imaging Center, Dijon, France; ^4^Department of Imaging, Turin Clinic, Paris, France; ^5^Centre d'Explorations Fonctionnelles Otoneurologiques, Paris, France

**Keywords:** endolymphatic hydrops, Meniere's disease, 3-Tesla MRI, vertigo, hearing loss

## Abstract

**Objective:**

The aim of this study was to compare different MRI diagnostic criteria for endolymphatic hydrops (EH) and to investigate the relation between audiovestibular and MRI findings in Meniere's disease (MD).

**Materials and methods:**

Prospective cross-sectional cohort study in 2 referral centers included 76 patients with unilateral (*n* = 62) or bilateral (*n* = 14) MD. All patients underwent inner ear 3T-MRI 4 h (*n* = 52) or >24H (*n* = 24) following audiovestibular tests. T2-CISS and 3D-FLAIR images 4H after gadolinium were obtained. EH diagnosis was based on saccular morphology on coronal views (T2 and 3D-FLAIR), semi quantitative estimation of endolymphatic space enlargement, and saccule utricle ratio inversion (SURI) on 3D-FLAIR axial views.

**Results:**

SURI was the best criterion related to the disease side (43 SURI+ on symptomatic ears, *n* = 77, *vs*. 6 SURI+ on asymptomatic ears, *n* = 53, *p* < 0.0001, Chi-2). Same-day MRI revealed relation between EH, hearing loss and caloric weakness which could not be detected on delayed MRI: SURI was associated with a higher pure-tone average (43 ± 4.1 dB in SURI+ ears, *n* = 42 *vs*. 23 ± 2.6 SURI-, *n* = 62, *p* < 0.0001, unpaired t-test,), and a higher proportion of vestibular caloric weakness (23/46 SURI+ ears *vs*. 4/62 SURI-, *p* < 0.001, Chi-2). Among all criteria, SURI combined to caloric weakness was the best predictor of the affected side in a logistic regression model.

**Conclusion:**

SURI had the strongest relation to the side the disease and audio vestibular findings for unilateral, probable and definite meniere disease. A short delay between MRI and audio vestibular tests improved the coherence between the findings.

## 1. Introduction

The relation between Meniere's disease (MD) and endolymphatic hydrops (EH) remains complex and unclear because MD is merely a syndrome (1), and its connection to EH as a pathophysiological entity is often hampered by incomplete features or other associations such as migraine (2). In many cases, MD is a provisional diagnosis when only cochlear or vestibular signs are present (1, 3). In these cases, several months or years of follow-up reveal the disease progression, and some-times, the diagnosis (3). Since the first reports of EH in temporal-bone specimens from patients with MD, it has been assumed that EH is the pathophysiological basis of the disease (4). Since it was impossible to visualize EH in living patients during several decades, many focused on tests such as wide-band tympanometry (5, 6) or electrocochleography (7–9) in a search for diagnostic and follow-up indicators, but false-positives and negatives obscured the relation between these indicators and the clinical signs.

By enabling the clinicians to finally see EH, 3-Tesla MRI was initially thought to simplify the task by clearly distinguishing the suffering ears from the healthy ones (4, 10). With time, different criteria and techniques have been suggested in recent years: different image sequences, slice orientations, and outcome measures (e.g., diameter and surface measurements, semi quantitative assessments, volumetric measurements) were reported to discriminate between symptomatic and normal ears (10–19). Indeed, this new tool confirmed the relation between EH and MD in the majority, but also revealed discrepancies in many cases (17, 18, 20). EH was observed in ears contralateral to the symptomatic side and even in individuals with no signs, confirming older histopathological observations (20, 21). Other patients with typical MD did not present with EH (17, 18). While false negatives were explained by the reversibility of the EH in some cases, the presence of EH in normal ears remains a challenging pathophysiological question (22).

We hypothesized that the diagnostic criteria on MRI, and the delay between audiovestibular tests and the imaging could be two factors which influence the correspondence between MRI and clinical or audiovestibular findings in detecting EH. Hence, the aim of this study was to evaluate the signs of EH on MRI conducted immediately after audiovestibular tests or on a different day, and to use different reported MRI criteria and techniques to analyze the correspondence between MRI and other routine indicators in MD.

## 2. Patients and methods

This prospective cross-sectional multicenter cohort study included 76 consecutive adult patients (> 18 years) diagnosed with a definite or probable Meniere's disease (MD) according to the international classification (1) and seen for the first time in two tertiary referral centers between June 2016 to July 2018. MD was defined as follows.

Definite MD (DMD):

A. Two or more spontaneous episodes of vertigo, each lasting 20 min to 12 h.
B. Audiometrically documented low- to medium-frequency sensorineural hearing loss in one ear, defining the affected ear on at least one occasion before, during or after one of the episodes of vertigo. The sensorineural hearing loss is defined by the Barany Society (1) as “Low-frequency sensorineural hearing loss is defined as increases in pure tone thresholds for bone-conducted sound that are higher (i.e.,worse) in the affected ear than the contralateral ear by at least 30 dB HL at each of two contiguous frequencies below 2,000 Hz”.
C. Fluctuating aural symptoms (hearing, tinnitus, or fullness) in the affected ear.
D. Not better accounted for by another vestibular diagnosis.

Probable MD (PMD):

A. Two or more episodes of vertigo or dizziness, each lasting 20 min to 24 h.
B. Fluctuating aural symptoms (hearing, tinnitus, or fullness) in the affected ear.
C. Not better accounted for by another vestibular diagnosis.
D. Patients were not selected based on their previous or ongoing MD treatments. Ongoing treatments were pursued, and no change or modification was imposed.

To investigate the effect of delay between audiovestibular tests and MRI on the correspondence of diagnostic signs between imaging and tests, audiovestibular investigations were followed by the cranial 3-Tesla MRI a different day in the 24 first consecutive cases (32%, interval: 38 ± 54.4 days, median: 16.5, range:1–203), or the same day (4 h later) in the next consecutive 52 cases (68%). Patients with other associated audiovestibular disorders were excluded from the study.

The study was strictly conducted according to a protocol approved by the institutional ethical committee (CPP Est I, number: 20016-A00875-46). Patients provided their oral and written consent.

### 2.1. Clinical and audiovestibular data

Clinical data regarding age, gender, duration of MD, vestibular drop attacks, cochlear signs (hearing fluctuations, tinnitus, fullness), vestibular signs (vertigo attacks, average frequency during the last 6 months, duration) were systematically recorded.

Audiometry comprised pure-tone average (PTA, 0.5–4 kHz), and dissyllabic (French Fournier lists) word discrimination score (WDS). Audiometries (AC40^®^, Interacoustics, Middelfart, Danmark) were conducted in a standard audiometry booth with a headphone. Electrocochleography (ECoG) was measured by an extratympanic electrode (Elios^®^, Echodia^®^, Clermont-Ferrand, France) and averaging the response to 1,000 clicks at 85 dB HL. Action (AP) and summation potential (SP) amplitudes were recorded. SP/AP > 0.33 was considered in favor of a hydrops (9). Bithermal caloric vestibular testing vestibular test was performed by water irrigation in a calibrated setting at 30 and 44°C and automatic videonystagmography (Visual Eyes Spectrum^®^, Interacoustics, Middelfart, Danmark). For patients with perforated tympanic membrane or tympanostomy tubes, caloric tests were performed with air and not with water in both ears. A caloric weakness was defined by a response asymmetry above 20% based on the maximal slow-phase velocity of the nystagmus according to Jongkees (23).

Cervical vestibular evoked myogenic potentials (cVEMP) were measured by 500 Hz tone bursts at 90–100 dB HL with the rate of 7.1 stimuli/s transmitted *via* inserted headphones (Eclipse^®^, Interacoustics^®^). 100 stimuli were applied to each ear. The rise/fall time was 1 ms and plateau time 2 ms. cVEMP amplitudes were corrected for muscular pre-tension of the sternocleidomastoid muscle and patients with an air-bone gap at 500 Hz were excluded from cVEMP measurements. Four electrodes were required for the cVEMP recording: a negative electrode at the forehead, two positive electrodes at the junction of the upper and middle thirds of the sternocleidomastoid muscles and one ground electrode at the sternal manubrium. Contraction of the sternocleidomastoid muscle on the homolateral side was required. The patient was then asked to turn his head to the side opposite to the sound stimulation. P13 et N23 wave amplitudes (A, μV) were recorded and compared to the contralateral side. Asymmetry ratio was calculated by: (A_Unaffected_ – A_Affected_) / (A_Affected_ + A_Unaffected_). An absent response or an asymmetry > 0.35 was considered as a dysfunction (24). Other pathological test values were absent or increased latencies of early waves: a P13 wave appearing after 10 ms and/or an N23 wave arising after 19 ms. Video Head-Impulse test (vHIT, Otometrics^®^, Hoerskaetten, Danemark) provided vestibulo-ocular reflex (VOR) gains for each semicircular canal. Gains < 0.8 and the presence of corrective saccades were considered to indicate hypofunction of the respective canal (25). The disease side was defined by the cochlear signs including fluctuating hearing loss, fullness, and tinnitus. MD was considered as bilateral when cochlear signs involved both ears.

### 2.2. 3-Tesla magnetic resonance imaging

**Figure 1:**
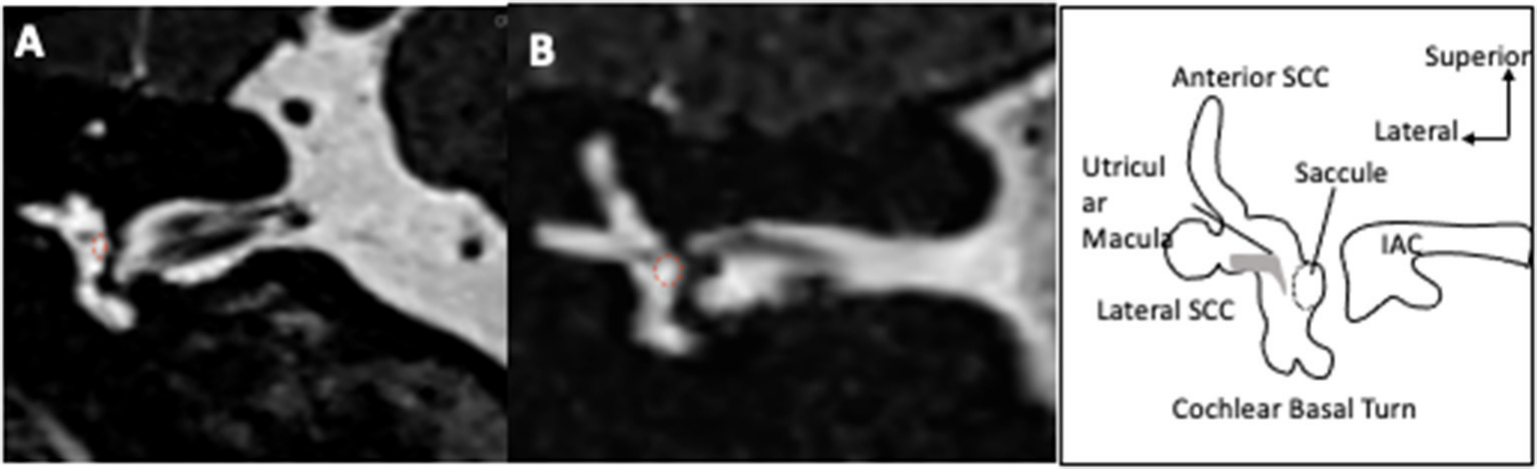
Visualization of saccule on MRI T2-weighted images. The coronal slices pass through the vestibule and show an oval shaped saccule in its superior exterior quadrant in an ear with no hydrops **(A)**. In case of hydrops **(B)**, the saccule is larger and approaches a circular shape. The saccule is surrounded by a dotted red circle.

High-resolution T2- weighted images (3D SPACE) of both inner ears were obtained in all 76 cases (Siemens 3 Tesla^®^ SPECTRA) with a head antenna. In addition, 3D-FLAIR images 4H after intravenous injection of gadolinium (0,2 ml/kg of gadoteric acid, Dotarem^®^ at 0,5 mmol/ml) were available in 68 patients (same day as audiovestibular tests in 54 and a different day in 14 cases). T2-sequence-weighted images had the following characteristics: slice thickness 0.3 mm, 1,200 ms relaxation time, 145 ms echo time, number of excitations: 2, 320x356 Matrix, 120° Flip angle, acquisition time:12,269 ms. The saccule was visualized on the coronal views (Figure 1) and its maximal height and width were measured (Osirix v.5.6^®^, Pixmeo^®^, Bernex, Suisse). Hydrops was defined by a height > 1.6 mm and/or a height/width ratio < 1.14 with a tendency toward a round saccule (saccule height and width criteria) (11). All 90 ears had a T2-weight CISS sequence. However, the fuzzy contours of the saccule in this sequence did not allow to measure all parameters with certainty. Consequently, 80 or 88 cases depending on the parameter could be reported.

**Figure 2:**
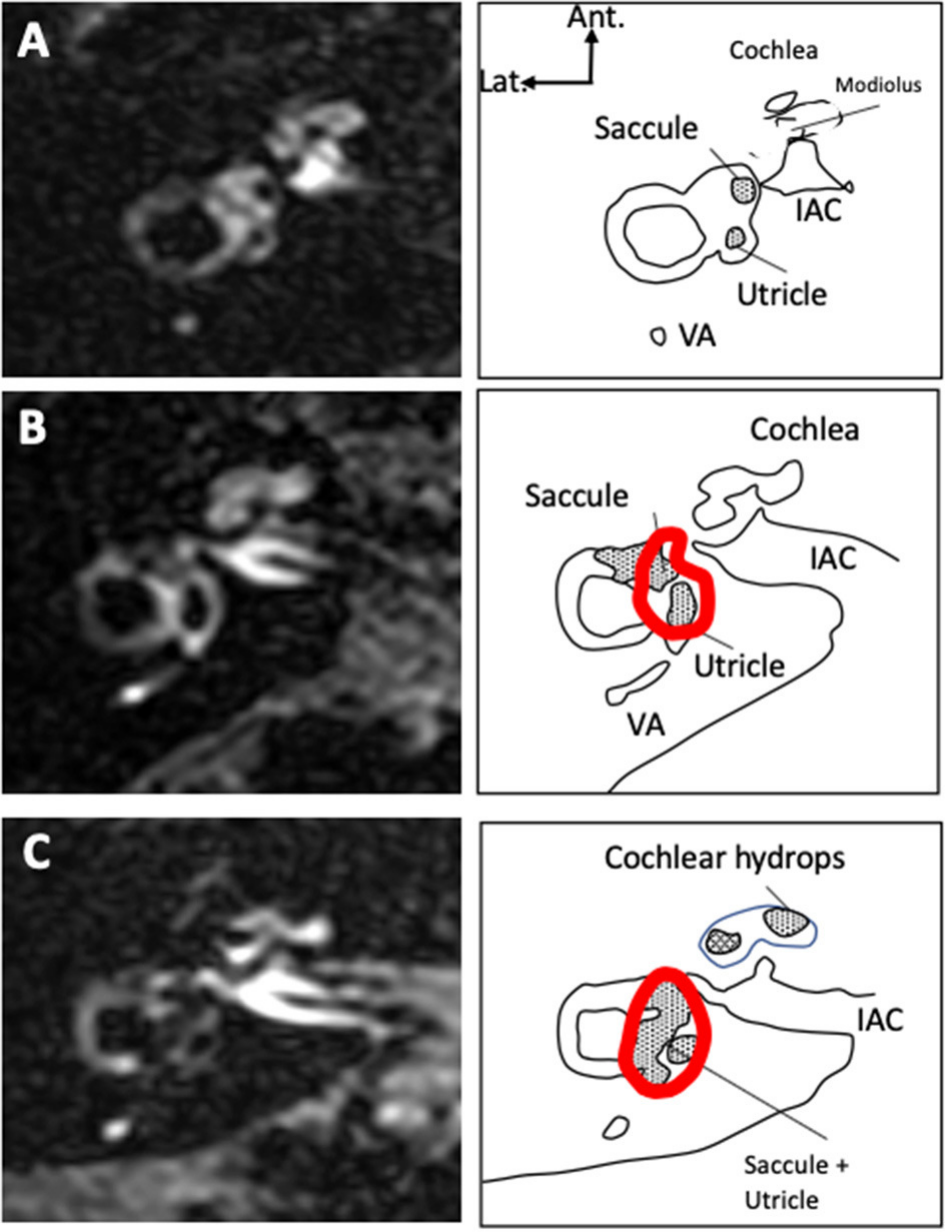
Vestibular and cochlear hydrops on 3D-FLAIR axial views 4 hours after IV gadolinium. Examples of ears with no signs of hydrops [grade 0, **(A)**], with moderate hydrops [grade I vestibular and cochlear hydrops, **(B)**], and significant hydrops [grade II vestibular and grade I cochlear hydrops, **(C)**] are presented. The total surface of the vestibule as measured in the study is contoured in red. Only the saccular surface inside the vestibular area was measured. In case of extensive hydrops **(C)**, the saccule could not be distinguished from the utricle and the surfaces of both structures were measured together.

**Figure 3:**
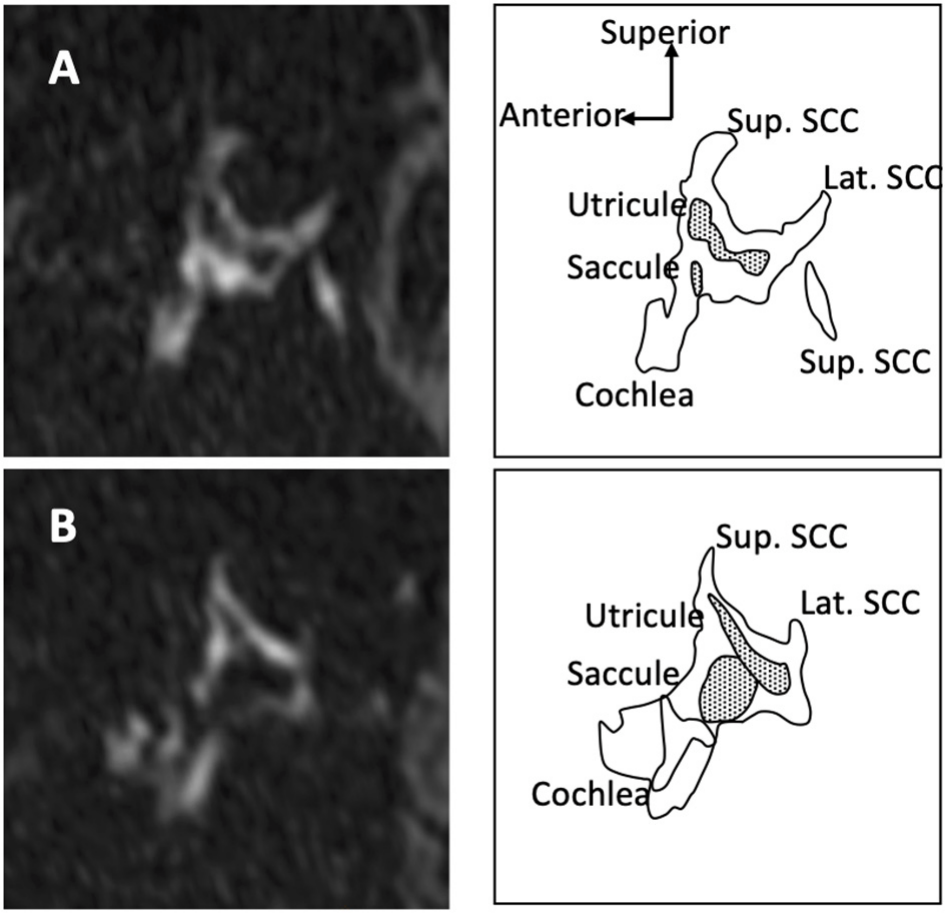
Saccule and utricle on 3D-FLAIR oblique sagittal views. When there is no hydrops **(A)** saccule appears smaller than utricle. In case of hydrops **(B)**, the saccule becomes larger than the utricle and a saccule/utricle ratio inversion (SURI) can be observed.

3D-FLAIR images were obtained 4H after gadolinium with the following parameters: slice thickness 0.3 mm, 8,000 ms relaxation time, 498 ms echo time, 2,350 ms inversion time, number of excitations: 2, 320x320 Matrix, variable Flip angle, and 8:58 min acquisition time. On an axial view at the level of the lateral semicircular canal, the entire surface of the vestibular endolymphatic compartment (saccule+utricle) and entire vestibular surface (perilymphatic+endolymphatic compartments) were estimated by manual contouring and the endolymphatic surface/entire vestibule surface was deduced (Figure 2). The contouring was performed on only one slice at the level of the lateral semicircular canal. Vestibular hydrops (VH) was graded as 0 (no hydrops) for a ratio < 0.33, as I (moderate) for ratios 0.33–0.49, and II (significant) for ratios 0.5–1.0 according to Nakashima et al. (10). On a view at the level of modiolus, we graded the cochlear hydrops (CH) as 0 (no hydrops) when no dilatation of the cochlear canal was detected, as I (moderate) when a moderate dilatation of the cochlear canal coexisted with a visible vestibular scala, and as II (significant) when the dilatation of the cochlear canal totally masked the vestibular ramp (axial surface ratio criteria) (17). On the coronal plane, the saccule was measured similarly to T2-view analysis, and the same diagnostic criteria were applied. Finally, on the sagittal plane, we identified both the utricle and the saccule in their greatest diameters on the same view. We estimated their surfaces by manual contouring and deduced the saccule/utricle ratio. A ratio > 1 was considered as a saccule/utricle ratio inversion (SURI) indicative of an EH (Figure 3) (18, 26).

MRI scans were obtained in two centers (39 in center 1, and 37 in center 2) and analyzed by one of the radiologists for each patient (ED for center 1, and JLB for center 2). Measurements and gradings were conducted according to the preestablished protocol. Radiologists were aware of the suspected diagnosis but were blinded regarding the side. Age, sex ratio, duration of symptoms before investigation, DMD/PMD ratio did not differ between the two centers (mean age: 51.2 ± 15.26 years, median: 51.0, range 21–78 *vs*. 57.4 ± 14.23, median: 59.0, range: 27–78, *p* = 0.08; sex ratio 0.44 *vs*. 0.85, *p* = 0.24; duration of symptoms 71.7 ± 71.94 months, median: 49.0, range: 0–272 *vs*. 89.0 ± 92.29, median: 62, range: 2–368, *p* = 0.36, Mann-Whitney test; DMD/PMD ratio 1.6 *vs*. 4.3, Chi-2 test, *p* = 0.08 for centers 1 *vs*. 2 respectively).

### 2.3. Data management and analysis

Data was collected and managed by Excel^®^ (Microsoft 2010, Microsoft Inc. Redmond, VI, USA), and analyzed by Prism^®^ (v.6, Graphpad Inc., San Diego, CA, USA). Most continuous variables did not pass D'Agostino and Pearson's test for normal distribution. Consequently, they were compared by a Mann-Whitney non-parametric test between two unpaired groups, Kruskal-Wallis test for more than 2 unpaired groups or a Wilcoxon signed-rank test in case of paired comparisons. Nominal variables were compared by a Chi-2 test. Correlations between continuous variables were evaluated by the Spearman coefficient and a F-test. R2 > 0.5 with *p* < 0.05 were considered as significant. Inter criterion reliability was tested by Cohen's kappa on XLSTAT^®^ (Addinsoft, New York, NY, USA). Logistic regression analysis was conducted on Statview software^®^ (v.6, SAS Inc., Cary, NC, USA). Values were presented as means ± standard deviation (SD), n, median and range. Supporting data is available at an online repository: Bozorg Grayeli, Alexis (2022), Confrontation of Endolymphatic Hydrops Diagnosis on 3-Tesla MRI to Clinical and Audiovestibular Findings in Meniere's Disease, Dryad, Dataset, https://doi.org/10.5061/dryad.hqbzkh1j7.

## 3. Results

### 3.1. Population

The population comprised 47 women (62 %) and 29 men (38 %). The mean age was 54 ± 1.7 years (range: 21–78). Fifty-four patients (61%) had a DMD, and 22 (29%) had a PMD. Sixty-two patients (81%) had a unilateral MD (UMD, 30 right, 32 left), and 14 (19%) had a bilateral disease (BMD). The disease duration at the time of inclusion was 6.2 ± 6.79 years (median: 4, range: 0.1–30). The average number of vertigo spells per month was 4.4 ± 8.85 (median: 1, range: 0.1–30) and the duration of each episode was estimated as 16.3 ± 23.51 h (median: 5, range: 0.1–96). Nine patients (12%) reported vestibular drop attacks. An ongoing medical treatment was noted at the time of inclusion in 51 patients (67%). The treatment was oral glycerol in 16 (21%), betahistine in 14 (18%), tympanostomy tube in 5 (7%), *in situ* gentamycine in two cases (3%), acetazolamide in 1 (1%), and other symptomatic vertigo medications in 38 patients (50%). Among these, 22 declared an improvement after the treatment onset (44%). Patients with an ongoing treatment at inclusion were predominantly female (sex ratio: 0.4, *n* = 49 *vs*. 1.25, without treatment), but they did not differ from those without a treatment in terms of age (53.2 ± 15.92 years, median 53, range: 21–78 *vs*. 56.0 ± 13.23, median 58, range: 28–73, respectively, *p* = 0.46, Mann-Whitney test), frequency of vertigo spells (5.6 ± 10.02 /month, median: 1, range: 0–30 *vs*. 2.4 ± 6.02, median: 0.5, range: 0–30, respectively, *p* = 0.16, Mann-Whitney test), duration of vertigo spells (14.2 ± 19.28 hours, median: 5, range: 0–96, *vs*. 19.9 ± 29.60, median: 3, range: 0.02–96, respectively, *p* = 0.72, Mann-Whitney test), proportion of cases with hearing fluctuation in the involved ears (61%, *n* = 59 *vs*. 70%, *n* = 30, respectively, *p* = 0.40), and PTA in the involved ears (42.5 ± 26.99 dB HL, median: 41.9, range: 5–120, *n* = 58 *vs*. 39.2 ± 27.68, median: 33.8, *n* = 29, respectively, *p* = 0.55).

In DMD, 63 ears were affected among 108 (58%), while in PMD, 27 ears out of 44 were symptomatic (62%).

The mean age was higher in DMD than in PMD (56.3 ± 14.94 years, median: 56, range: 21–78 *vs*. 48.9 ± 14.81, median: 49, range: 21–70, respectively, *p* < 0.01, Mann-Whitney test). The sex ratio was also different between the groups with a male predominance in DMD (0.86 in DMD *vs*. 0.22 PMD, *p* = 0.0066, Fisher's exact test). The disease duration at the time of inclusion was significantly longer in DMD than in PMD (7.1 ± 7.05 years, median: 5, range: 0.1–30 *vs*. 4.2 ± 5.68, median: 2, range: 0.1–21 respectively, *p* = 0.0043, Mann-Whitney test).

### 3.2. Audiovestibular findings

Expectedly, the hearing loss dominated on the involved ear in cases UMD; and it was greater on the right side in our BMD patients (Table 1). As expected, ECoG was bilaterally abnormal in most of BMD, but also in most patients with UMD. Average SP/AP ratios did not differ between ears in both UMD and BMD groups (Table 1).

**Table 1:** Audiovestibular findings in unilateral (UMD) and bilateral Meniere's diseases (BMD).

	**UMD**			**BMD**		
	**Ipsilateral**	**Contralateral**	***p-v*** **alue**	**Right**	**Left**	***p-v*** **alue**
PTA (dB HL)	44 ± 28.6 (62)	26 ± 29.1 (62)	**< 0.0001**	44 ± 26.6 (14)	27 ± 17.6 (14)	**0.0499**
	43,8 [6-120]	16.3 [0-100]		36.9 [5-91.2]	26.3 [5-63.8]	
Caloric weakness	27/59 (44%)	7/59 (13%)	**< 0.0001**	5/13 (38%)	5/13 (38%)	1.0
cVEMPs	14/49 (29%)	7/49 (14%)	0.0848	2/11 (18%)	3/11 (27%)	0.4749
SP/AP	0.7 ± 0.77 (54)	0.6 ± 0.50 (54)	0.7034	0.6 ± 0.41 (14)	0.6 ± 0.51 (14)	0.5525
	0.53 [0-4.17]	0.62 [0-2.76]		0.60 [0-1.37]	0.48 [0-2.08]	
Abnormal ECoG (SP/AP>0.33)	29 bilateral + 7 ipsi. only/54	29 bilateral + 7 contra. only/54	1.0	7 bilateral /14	7 bilateral + 2 left only /14	0.4450

Caloric weakness was defined by asymmetrical caloric response > 20% according to Jongkees (23). Continuous variables are presented as mean ± standard deviation (n), median and [min.-max.]. PTA, Audiometric pure-tone average; SP/AP, summation potential/action potential on ECoG. p-values expressed for comparison vs. opposite ear, Wilcoxon test for continuous and Chi-2 for categorical parameters.

Caloric tests in UMD and BMD patients were not always in accordance with the disease side, and in UMD patients, even contralateral weakness could be detected (Table 1). Like the caloric test, cVEMP revealed both relative reduction of amplitudes on the ipsi- or the contralateral side involvement in UMD patients. In the BMD group, only a unilateral involvement was reported in 5 cases. vHIT did not show an abnormal gain in this population (average gain in horizontal plane 0.99 ± 0.09, median: 1.0, range: 0.83–1.16 for right and 0.94 ± 0.09, median: 0.96, range: 0.75–1.06 for left in BMD, *n* = 11, 0.89 ± 0.22, median: 0.95, range: 0.26–1.36 for ipsi- and 0.92 ± 0.22 for contralateral, median: 1.00, range: 0.15–1.37, *n* = 54 in UMD).

In DMD, audiologic findings and cVEMP indicated a more severe inner ear dysfunction in comparison to PMD (Table 2). In accordance with audiovestibular tests, MRI showed cochlear and vestibular hydrops more frequently in DMD than in PMD (Table 2).

**Table 2:** Audiovestibular and MRI findings in symptomatic ears with definite (DMD) *vs*. probable Meniere's disease (PMD).

	**DMD**	**PMD**	***P-v*alue**
**Audiovestibular findings**			
PTA (dB HL)	46.3 ± 22.72 (63)	29.8 ± 6.47 (27)	**0.0008**
	41.2 [5–120]	15.0 [5–120]	
Caloric weakness	31/52 (59%)	8/25 (32%)	0.27
cVEMPs	12/51 (24%)	0/21	**0.015**
SP/AP	0.72 ± 0.78 (57)	0.49 ± 0.32 (25)	0.49
	0.55 [0–4.17]	0.51 [0–1.15]	
Abnormal ECoG (SP/AP>0.33)	41/57 (72%)	17/25 (68%)	0.72
**FLAIR-3D 4H after Gadolinium**			
Total vestibular surface (axial)	19.4 ± 2.72 (53)	17.7 ± 0.51 (24)	0.052
	19.5 [11.7–26.0]	17.4 [12.8–24.9]	
Vestib. endolymhatic surface (axial)	5.8 ± 1.74 (50)	4.1 ± 1.31 (22)	**0.0003**
	5.8 [3.0–11.2]	4.2 [1.4–6.6]	
Endolymphatic surface/Total surface	0.30 ± 0.93 (50)	0.24 ± 0.79 (22)	**0.0055**
	0.3 [0.2–0.5]	0.2 [0.1–0.4]	
Vestibular hydrops grades n (0/I/II)^£^	30/19/1	20/2/0	**0.0314**
Cochlear hydrops grades n (0/I/II)^£^	24/26/3	19/5/0	**0.0181**
Saccular surface (sagittal)	3.2 ± 1.40 (53)	2.0 ± 1.14 (24)	**0.0004**
	3.4 [0.9–6.3]	1.4 [0.6–4.7]	
Utricular surface (sagittal)	2.9 ± 1.14 (53)	3.3 ± 1.00 (24)	0.1362
	2.8 [1.2–6.6]	3.2 [1.4–4.5]	
Saccule/Utricule (sagittal)	1.3 ± 0.69 (53)	0.7 ± 0.56 (24)	**0.0015**
	1.2 [0.2–3.0]	0.5 [0.1–2.1]	
Hydrops n/all cases (SURI)	36/53 (68%)	7/24 (29%)	**0.0015**
Saccular Height (coronal)	2.2 ± 0.59 (53)	1.8 ± 0.42 (24)	**0.0019**
	2.1 [1.2–4.4]	1.8 [1.1–3.0]	
Saccular Width (coronal)	1.2 ± 0.34 (53)	1.1 ± 0.26 (24)	0.1906
	1.1 [0.7–2.0]	0.98 [0.8–1.8]	
Saccular Height/Width	2.0 ± 0.56 (53)	1.8 ± 0.61 (24)	0.1048
	2.0 [1.0–3.2]	1.8 [0.9–3.8]	
Hydrops (Height/Width) ^££^ n/total	49/53 (92%)	18/24 (75%)	**0.0348**
**T2-weighed CISS**			
Vestibular Surface (axial)	16.4 ± 2.25 (61)	15.9 ± 2.10 (27)	**0.3537**
	16.2 [12.4–23.4]	15.9 [12.4–19.4]	
Saccular Height (coronal)	1.9 ± 0.48 (56)	2.0 ± 0.38 (24)	0.3447
	1.8 [1.0–3.0]	2.0 [1.4–3.3]	
Saccular Width (coronal)	1.3 ± 0.29 (56)	1.4 ± 0.41 (24)	0.2635
	1.3 [0.8–2.5]	1.4 [0.9–2.5]	
Height/Width	1.49 ± 0.40 (56)	1.45 ± 0.28 (24)	0.6822
	1.5 [0.8–2.6]	1.4 [1.0–2.2]	
Hydrops (Height and Width) ^£^ n/all cases	49/56 (88%)	21/24 (88%)	1.0

Caloric weakness was defined by asymmetrical caloric response >20% according to Jongkees (23). Lengths are expressed in mm and surfaces in mm^2^. EL, endolymphatic; SURI, Saccule/Utricle ratio inversion.

Vestibular and cochlear gradings according to Nakashima et al. (10); and Barath et al. (17).

££ Hydrops was defined by a height > 1.6 mm or a height/width ratio < 1.14 (11). Continuous variables are presented as mean ± standard deviation (n), median [min.-max.]. PTA, Audiometric pure-tone average; n, number of available tests, p-values are reported for DMD vs. PMD comparisons with Mann-Whitney for continuous variables Chi-2 test for categorical parameters.

### 3.3. 3D-FLAIR images

The proportion of VH based on semi quantitative assessment of the same parameters was not different between affected and asymptomatic ears (Table 3) or between sides in patients with UMD (Table 4). However, in UMD, both the vestibular endolymphatic surface and the saccule surface (in sagittal plane) were higher on the disease side (Table 4). The proportion of EH based on height and width were also similar in both groups except for the saccular width (coronal plane) which was higher for symptomatic ears *vs*. asymptomatic ears and for the ipsilateral ears *vs*. contralateral ears in patients with UMD (Tables 3, 4). Paradoxically, saccular height/width ratio was not modified between the two ears in patients with UMD (Table 4). In sagittal plane, saccular surfaces were higher for symptomatic ears than asymptomatic ears and utricular surfaces were lower for symptomatic ears than asymptomatic ears (Table 3). In BMD, PTA showed a dominant loss on the right side (Table 1). According to 3D-Flair images results (no difference between right and left sides except the saccule surface in sagittal plane), EH did not seem to influence the PTA in patients with BMD (Table 4).

**Table 3:** MRI findings in symptomatic *vs*. asymptomatic ears.

**FLAIR-3D 4H after Gadolinium**	**Symptomatic ears**	**Asymptomatic ears**	***p-v*alue**
Total vestibular surface (axial)	18.9 ± 2.75 (77)	18.4 ± 3.01 (53)	0.2962
	18.7 [11.7–26]	17.9 [12.4–24]	
Vestib. endolymphatic surface (axial)	5.3 ± 1.80 (77)	4.7 ± 1.77 (52)	0.0579
	5.1 [1.4–11.2]	4.3 [1.5–8.5]	
Endolymphatic surface/Total surface	0.28 ± 0.094 (72)	0.25 ± 0.082 (52)	0.1207
	0.28 [0.08–0.53]	0.25 [0.09–0.44]	
Vestibular hydrops grades n (0/I/II) ^£^	50/21/1	44/8/0	0.1293
Cochlear hydrops grades n (0/I/II) ^£^	43/31/3 ^$^	42/11/0	**0.0151**
Saccular surface (sagittal)	2.8 ± 1.43 (77)	1.9 ± 1.13 (53)	**< 0.0001**
	2.5 [0.6–6.3]	1.6 [0.7–7.0]	
Utricular surface (sagittal)	3.0 ± 1.11 (77)	3.5 ± 1.16 (53)	**0.0147**
	2.9 [1.2–6.6]	3.4 [1.1–6.3]	
Saccule/Utricule (sagittal)	1.1 ± 0.69 (77)	0.6 ± 0.49 (53)	**< 0.0001**
	1.1 [0.1–3.0]	0.44 [0.2–2.7]	
Hydrops n/all cases (SURI)	43/77 (56%)	6/53 (11%)	**< 0.0001**
Saccular Height (coronal)	2.1 ± 0.58 (77)	2.0 ± 0.46 (53)	0.4192
	2.0 [1.1–4.4]	2.0 [1.1–3.6]	
Saccular Width (coronal)	1.1 ± 0.32 (77)	1.0 ± 0.28 (53)	**0.0498**
	1.0 [0.7–2.0]	1.0 [0.6–1.9]	
Saccular Height/Width	1.9 ± 0.58 (77)	2.0 ± 0.56 (53)	0.2615
	1.9 [0.9–3.8]	2.0 [0.9–3.3]	
Hydrops (Height/Width) ^££^ n/total	67/77 (87%)	44/53 (83%)	0.5264
**T2-weighed CISS**			
Vestibular Surface (axial)	16.2 ± 2.21 (88)	16.2 ± 2.15 (60)	0.9720
	16.2 [12.4–23.4]	16.2 [11.8–23.7]	
Saccular Height (coronal)	1.9 ± 0.45 (80)	2.0 ± 0.39 (56)	0.1913
	2.0 [1.0–3.3]	2.0 [1.2–3.0]	
Saccular Width (coronal)	1.3 ± 0.30 (80)	1.3 ± 0.28 (56)	0.3050
	1.3 [0.8–2.5]	1.28 [0.8–2.5]	
Height/Width	1.48 ± 0.37 (80)	1.6 ± 0.36 (56)	**0.0274**
	1.5 [0.8–2.6]	1.5 [0.9–2.4]	
Hydrops (Height/Width) ^£^ n/total	70/80 (88%)	52/62 (84%)	0.3116

Lengths are expressed in mm and surfaces in mm^2^. Continuous variables are presented as mean ± standard deviation (n), median, [min.-max.]. EL, endolymphatique; SURI, Saccule/Utricle ratio inversion.

£ Vestibular and cochlear gradings according to Nakashima et al. (10); and Barath et al. (17).

££ Hydrops was defined by a hight > 1.6 mm or a height/width ratio < 1.14 (11), Paired t-test vs. opposite ear, ^$^ p = 0.07 Chi-2 test.

**Table 4:** 3D-FLAIR MRI images in unilateral (UMD) *vs*. bilateral Meniere's Disease (BMD).

**Parameters**	**UMD**			**BMD**		
	**Ipsilateral**	**Contralateral**	***p-v*** **alue**	**Right**	**Left**	***p-v*** **alue**
Tot Vest. Surf. axial	18.7 ± 2.84 (53), 18.6 [11.7-26.0]	18.4 ± 3.02 (53), 17.9 [12.4-24.1]	0.4816	19.2 ± 2.52 (12), 18.9 [15.6-22.7]	19.4 ± 2.7 (12), 19.8 [12.8-22.8]	0.5829
Vest. EL Surf. axial	5.4 ± 1.91 (51), 5.2 [1.4-11.2]	4.7 ± 1.77 (52), 4.3 [1.5-8.5]	**0.0210**	5.1 ± 1.75 (12), 4.8 [2.6-7.7]	4.0 ± 2.23 (12), 4.1 [0-6.5]	0.2477
EL/Total Surf.	0.29 ± 0.10 (51), 0.28 [0.08-0.53]	0.25 ± 0.08 (52), 0.25 [0.09-0.44]	0.0672	0.27 ± 0.10 (12), 0.29 [0.13-0.43]	0.21 ± 0.13 (12), 0.22 [0.0-0.36]	0.2664
Vest. Hyd. n(0/I/II)^£^	35/15/1	44/8/0	0.7989	8/4/0	9/2/0	0.6576
Coch. Hyd. n(0/I/II)^£^	23/27/3	42/11/0	0.4586	11/1/0	9/3/0	0.0704
Saccule Surf. sagittal	2.9 ± 1.43 (53), 2.9 [0.6-6.3]	1.9 ± 1.13 (53) 1.6 [0.7-7.0]	**0.0001**	3.1 ± 1.47 (12), 3.0 [1.3-5.4]	1.9 ± 1.1 (12), 1.4 [1.1-4.2]	**0.0342**
Utricule Surf. sagittal	2.9 ± 1.04 (53), 2.8 [1.2-5.7]	3.5 ± 1.16 (53), 3.4 [1.1-6.3]	**0.0060**	3.3 ± 1.4 (12), 3.2 [1.3-6.6]	3.1 ± 1.1 (12), 2.8 [1.6-4.6]	0.4802
Sac./Utric. sagittal	1.2 ± 0.65 (53), 1.2 [0.1-3.0]	0.6 ± 0.49 (53), 7.5 [0.2-2.7]	**< 0.0001**	1.2 ± 0.84 (12), 1.0 [0.4-3.0]	0.8 ± 0.69 (12), 0.50 [0.3-2.5]	0.1579
SURI	35 (66%)	6 (11%)	0.0724	6 (50%)	2 (17%)	1.0
Sac. height coronal	2.1 ± 0.55 (53), 2.1 [1.2-4.4]	2.0 ± 0.46 (53), 2.0 [1.1-3.6]	0.0735	1.8 ± 0.52 (12), 1.8 [1.1-3.0]	2.2 ± 0.68 (12), 2.1 [1.2-3.4]	0.0597
Sac. width coronal	1.2 ± 0.35 (53), 1.1 [0.7-2.0]	1.0 ± 0.28 (53), 1.0 [0.6-1.9]	**0.0216**	1.1 ± 0.26 (12), 1.0 [0.9-1.7]	1.1 ± 0.23 (12), 1.0 [0.8-1.6]	0.1973
Sac. Height/width	2.0 ± 0.59 (53), 2.0 [0.9-3.8]	2.0 ± 0.56 (53), 2.0 [0.9-3.3]	0.4775	1.6 ± 0.50 (12), 1.6 [1.0-2.7]	2.1 ± 0.55 (12), 2.1 [1.1-2.8]	0.0597
Hydrops^££^ (n, %)	46 (87%)	44 (83%)	0.3807	11 (92%)	10 (83%)	0.6404

Among 76 patients, only 65 were assessed by this method. Lengths are expressed in mm and surfaces in mm^2^. Continuous variables are presented as mean ± standard deviation (n), median, [min.-max.]. Vest, Vestibular; Surf, surface; EL, endolymphatic; Sac., saccular; Utric., utricular; SURI, saccule/utricle ratio inversion.

£ Vestibular and cochlear gradings according to Nakashima et al. (10); and Barath et al. (17).

££ Hydrops was defined by a height > 1.6 mm or a height/width ratio < 1.14 (11), p-values are expressed for Wilcoxon's signed-rank or Chi-2 test vs. opposite ear.

The frequency of vertigo attacks was similar in VH grades 0 and 1 (3.2 ± 7.03 attacks/month, *n* = 87 *vs*. 6.0 ± 10.65, *n* = 27, respectively, *p* = 0.03, Mann-Whitney test). The duration of the disease was higher in ears with VH grade I or II than with grade 0 (93.5 ± 81.16 months, for grade I *vs*. 64.6 ± 72.31, *n* = 94, for grade 0, *p* = 0.035, Mann-Whitney test).

The frequency of attacks was not related to CH grades (4.0 ± 8.38 attacks/month, *n* = 79 for grade 0, *vs*. 3.2 ± 7.14, *n* = 38 for grade I, and 11.7 ± 15.95, *n* = 3 for grade II, respectively, *p* = 0.14, Kruskal-Wallis test). Also, the disease duration was not related to CH grades (75.0 ± 84.4 months, *n* = 85 for grade 0, *vs*. 76.6 ± 70.7, *n* = 42 for grade I, and 53.7 ± 31.3, *n* = 3 for grade II, respectively, *p* = 0.55, Kruskal-Wallis test).

Hearing loss seemed to be influenced by CH grades (PTA=61.3 ± 34.26 dB, *n* = 3 in grade II *vs*., PTA=43.3 ± 29.94 dB, *n* = 44 in grade I *vs*. 30.2 ± 27.59 dB, *n* = 84 in grade 0, *p* = 0.016, Kruskal-Wallis test).

Among selected criteria, SURI seemed to be best related to the side of the disease (Tables 3–5). The disease duration did not differ between ears with and without SURI (75.5 ± 80.89 months, *n* = 43 *vs*. 85.0 ± 80.63, *n* = 34, *p* = 0.86, Mann-Whitney test), but ears with SURI tended to be associated to more frequent attacks (3.4 ± 7.64 attacks/month, without SURI, *n* = 16 *vs*. 4.8 ± 9.12, with SURI, *n* = 33, *p* = 0.071, Mann-Whitney test).

**Table 5:** Relation between audiovestibular findings and endolymphatic hydrops on same-day and delayed MRIs.

	**Same-day MRI**			**Delayed MRI**		
**SURI**	+	−	***p-v*** **alue**	+	−	***p-v*** **alue**
PTA	43 ± 26.7 (42), 43 [3–100]	23 ± 20.3 (62), 16 [3–100]	**< 0.0001**	44 ± 33.8 (7), 49 [0–100]	53 ± 41.3 (17), 36 [9–120]	0.5893
Caloric	23/46	4/62	**< 0.0001**	4/5	1/12	0.0776
cVEMPs	6/40	7/60	0.6273	3/5	0/15	**0.0011**
ECoG	30/42	46/62	0.7551	3/7	6/15	0.4520
**FLAIR Vest. Surf**.	+	**–**	***p-v*** **alue**	+	**–**	***p-v*** **alue**
PTA	45 ± 27.4 (23), 41 [5–100]	27 ± 23.3 (75), 16 [3–100]	**0.0020**	52 ± 28.3 (6), 52 [15–100]	50 ± 42.3 (18), 36 [0–120]	0.6407
Caloric test	10/23^*^	15/75	**0.0238**	5/6	5/16	**0.0248**
cVEMPs	5/22	2/9	0.1097	2/6	1/14	0.1328
ECoG	17/23	56/75	0.9422	3/6	9/16	0.7932
**FLAIR** **Sac. H/W**	+	**–**	***p-v*** **alue**	+	**–**	**p** ***p-v*****alue**
PTA	31 ± 25.5 (86), 20 [3–100]	30 ± 23.9 (18), 26 [5–100]	0.8805	48 ± 37.4 (23), 36 [0–120]	110 (1)	0.1293
Caloric test	24/86	3/18	0.3226	10/21	0/1	0.3501
cVEMPs	11/82	2/18	0.7924	3/17	0/0	–
ECoG	62/86	14/18	0.6210	12/22	0/0	–
**T2** **Sac. H/W**	+	**–**	***p-v*** **alue**	+	**–**	***p-v*** **alue**
PTA	30 ± 24.2 (80), 20 [3–100]	42 ± 33.6 (11), 29 [6–100]	0.2759	47 ± 35.2 (39), 39 [0–120]	55 (1)	0.6337
Caloric test	19/80	5/11	0.1256	13/36	1/1	0.1938
cVEMPS	7/78	5/11	**0.0009**	4/18	0/1	0.5957
ECoG	57/80	10/11	0.1653	18/27	1/2	0.6323

Hydrops was defined on axial FLAIR-3D + gadolinium MRI views by saccule utricle ratio inversion (SURI), vestibular endolymphatic/total axial surface ratio (FLAIR Vest. Surf.), Saccular height and width criteria on coronal views (height > 1.6 mm or a height/width ratio < 1.14, FLAIR Sac. H/W), and on the same criteria on T2-weighed images (T2 Sac. H/W). For each criterion, audio vestibular test, fractions indicate the number of positive tests in favor of hydrops/total in each subgroup. Pure-tone average (PTA) is expressed as mean ± standard deviation (n), median, [min.-max.]. P-values are expressed for comparisons between hydrops (+) and no hydrops (−) by Mann-Whitney test for continuous variables and Chi-2 test for categorical parameters.

There was no significant correspondence between SURI and other MRI criteria based on FLAIR and T2-CISS images (Table 6), suggesting that each criteria evaluates a different aspect of the EH.

**Table 6:** Correspondence between SURI and other MRI criteria for endolymphatic hydrops in ears with definit (DMD) and probable (PMD) Menière's disease.

		**DMD**			**PMD**		
		**SURI** +	**SURI** −	***p-v*** **alue**	**SURI** +	**SURI** −	***p-v*** **alue**
T2 CISS Saccular Height/Width	+	33	35	0.1364	7	27	0.6072
	−	2	7		1	2	
FLAIR coronal Saccular Height/Width	+	38	41	0.0613	7	25	0.8498
	−	2	9		2	6	
FLAIR axial, vestibular hydrops grades	0	25	34	0.4399	9	26	0.3147
	1	13	13		0	3	
	2	1	0		0	0	
FLAIR axial, cochlear hydrops grades	0	19	33	0.0562	6	27	0.1556
	1	18	17		3	4	
	2	3	0		0	0	

Figures represent number of ears for each category. Total numbers vary between criteria due to missing values. P-values correspond to Chi-2 tests for comparison between SURI + (hydrops) and SURI–(no hydrops) for each criterion.

### 3.4. T2- weighted images

The proportion of hydrops according to height and width criteria was similar in clinically involved and unaffected ears except for the height/width ratio which was higher for the asymptomatic ears *vs*. symptomatic ears and for contralateral ears *vs*. ipsilateral ears in patients with UMD such as the saccular height in UMD (in the coronal plane, contralateral ears *vs*. ipsilateral ears) (Tables 3, 7). Similarly, Caloric test, cVEMP and ECoG did not seem related to EH detected on T2- weighted images. Finally, a same-day MRI did not produce a better correspondence between T2-MRI criteria and audiovestibular tests than later imaging expect for the correlation with cVEMP (Table 5).

**Table 7:** T2-weighed MRI images in unilateral (UMD) *vs*. bilateral Meniere's disease (BMD).

	**UMD**			**BMD**		
	**Ipsilateral**	**Contralateral**	***p-v*** **alue**	**Right**	**Left**	***p-v*** **alue**
Vest. Surf. axial	16.1 ± 2.20 (60), 16.1 [12.4-23.4]	16.2 ± 2.15 (60), 16.2 [11.8-23.7]	0.9158	16.5 ± 2.48 (14), 17.0 [12.9-20.2]	16.3 ± 2.10 (14), 16.4 [12.7-2.2]	0.6002
Sac. Height coronal	1.9 ± 0.43 (57), 1.9 [1.0-3.0]	2.0 ± 0.39 (56), 2.0 [1.2-3.0]	**0.0215**	1.8 ± 0.45 (12), 1.8 [1.3-2.7]	2.2 ± 0.51 (11), 2.2 [1.3-3.3]	0.0754
Sac. Width coronal	1.3 ± 0.29 (57), 1.3 [0.8-2.5]	1.3 ± 2.81 (56), 1.3 [0.9-2.4]	0.1515	1.2 ± 0.17 (12), 1.2 [0.8-1.4]	1.5 ± 0.37 (12), 1.5 [0.9-2.4] ^*^	**0.0033**
Height/Width	1.4 ± 0.38 (57), 1.4 [0.8-2.2]	1.6 ± 0.36 (56), 1.6 [0.9-2.4]	**0.0146**	1.6 ± 0.45 (12), 1.6 [0.9-2.6]	1.5 ± 0.15 (12), 1.6 [1.3-1.8]	0.3739
Hydrops ^£^ (n, %)	51/57 (89%)	52/56 (91%)	0.5266	9/12 (75%)	10/12 (83%)	0.6152

Lengths are expressed in mm and surfaces in mm^2^. Continuous variables are presented as mean ± standard deviation (n), median, [min.-max.]. Vest: vestibular, Surf: surface, EL: endolymphatic, Sac.: saccular, Utric: utricular,^£^Hydrops was defined by a height > 1.6 mm or a height/width ratio < 1.14 (11). P-values are expressed for comparisons between hydrops (+) and no hydrops (-) by Wilcoxon test for continuous variables and Chi-2 test for categorical parameters.

There was no agreement between saccular height-width criteria and any of the criteria on 3D-FLAIR to indicate the side of the involved ear (Cohen's kappa < 0.1). Similarly, there was no agreement between SURI and other criteria based on 3D-FLAIR views (saccular height and width on coronal views or endolymphatic surface on axial views, Cohen's kappa < 0.1). We observed a minimal accordance between SURI and the presence of CH (Cohen's kappa = 0.23), and between VH and CH grades on 3D-FLAIR views (Cohen's kappa = 0.18). Although total vestibular surfaces were well correlated between T2- weighted and FLAIR-3D sequences (Y=4.61+0.85X, Y: surface on FLAIR, X: surface on T2, R=0.65, *p* < 0.0001, F test), saccular height and width on T2 were not correlated to those on FLAIR-3D (R^2^ = 0.02, F = 1.47, *p* = 0.23 for the height and R^2^ = 0.07, F = 4.94, *p* = 0.03 for the width, *n* = 67, ANOVA).

### 3.5. Receiver operating characteristic curves for MRI diagnostic criteria

**Figure 4:**
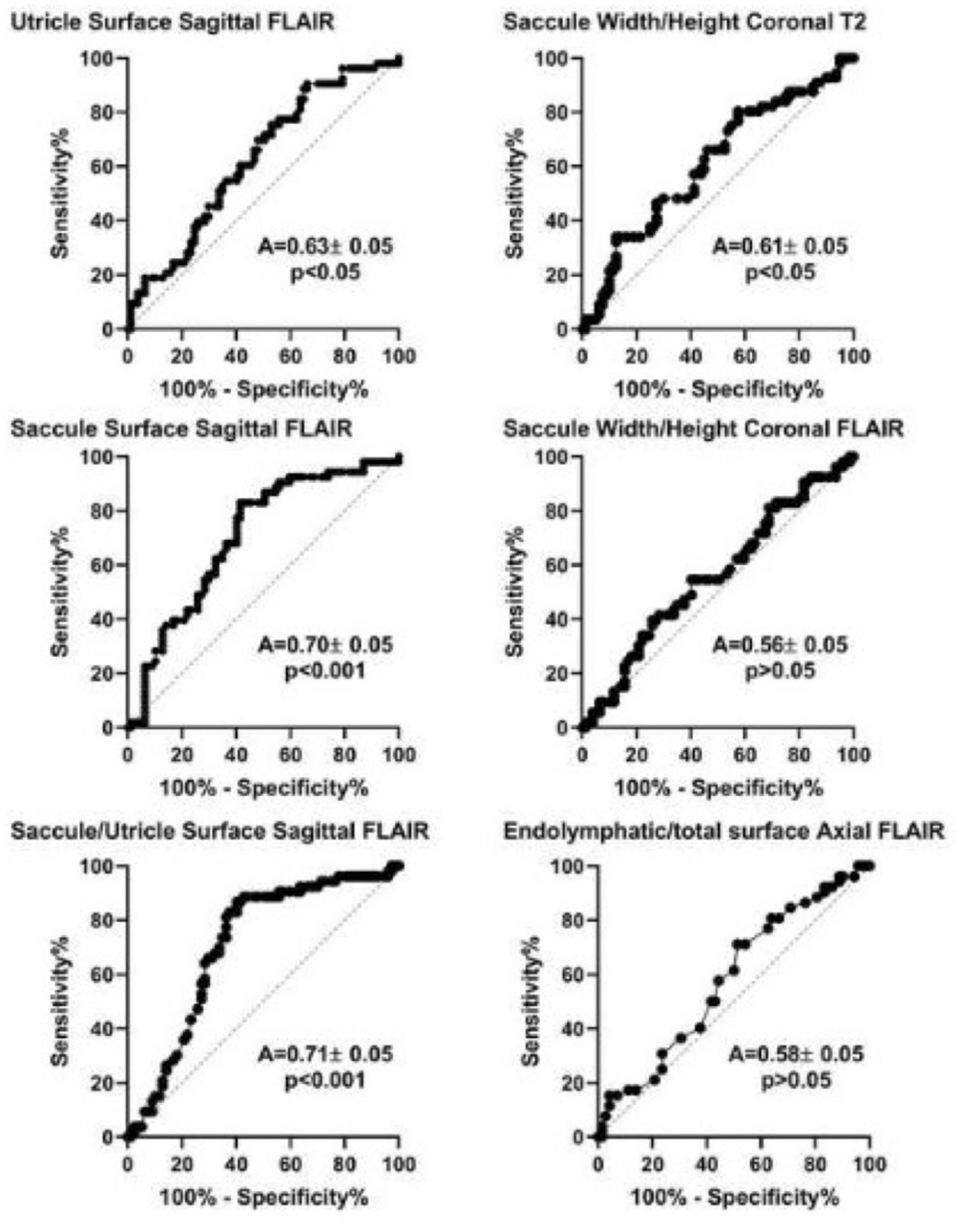
Receiver operation characteristic (ROC) analysis of different MRI diagnostic criteria based on the clinical diagnosis of Meniere's disease (international criteria). A, area under the curve (mean ± standard error). *P-value* corresponds to one-sample test for comparison vs. 0.5.

Analysis of Receiver Operating Characteristic (ROC) curves showed that among MRI diagnostic criteria, saccule surface on sagittal views, saccule/utricle surface ratio on sagittal 3D-FLAIR views, and saccule height/width ratio on coronal T2 views could discriminate between asymptomatic and affected ears according to clinical criteria (Figure 4). Discrimination capacity of T2 views was low: a ratio < 1.14 had a sensitivity of 88% but a specificity of only 23% (likelihood ratio=1.13). The same parameter on 3D-FLAIR, and endolymphatic/total vestibular surface on axial views could not discriminate between involved and asymptomatic ears. In contrast, Saccule/Utricle surface > 1on sagittal 3D-FLAIR views yielded a sensitivity of 81% and a specificity of 64% (likelihood ratio = 2.2).

**Figure 5:**
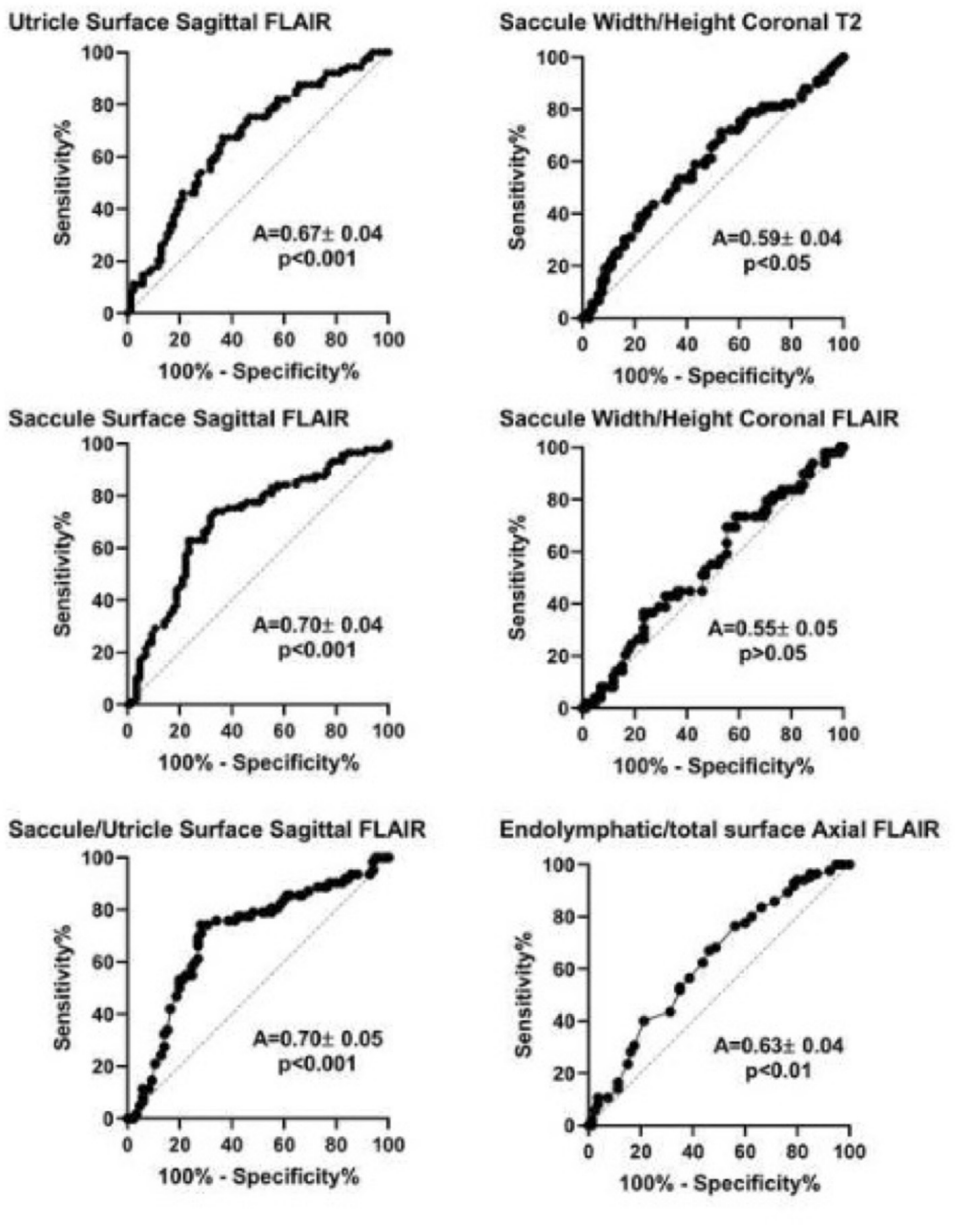
Receiver operation characteristic (ROC) analysis of different MRI diagnostic criteria based on vestibular caloric weakness in Meniere's disease. A, area under the curve (mean ± standard error). *P-value* corresponds to one-sample test for comparison *vs*. 0.5.

By choosing the caloric weakness as the indicator of the affected side instead of clinical criteria, not only height/width on T2 and saccule/utricle ratio on 3D-FLAIR but also relative endolymphatic surface on axial 3D-FLAIR appeared to indicate the affected side (Figure 5). Among these criteria, the saccule/utricle ratio on 3D-FLAIR had the best discrimination ability and detected the weak caloric side with a 74% sensitivity, a 72% specificity (likelihood ratio = 2.6).

By selecting SP/AP > 0.33 on EcoG as an indicator of the disease, no MRI diagnostic criteria could distinguish between involved and normal ears as judged by ROC analysis (Table 8).

**Table 8:** Performance of MRI criteria by ROC analysis to distinguish between involved and normal ears.

**MRI Criteria**	**EcoG** **(SP/AP>0.33)**	**EcoG/MRI same day** **(SP/AP>0.33)**
Utricle surface sagittal FLAIR	AUC= 0.52, *p =* 0.72	AUC= 0.63, ***p** **=*** **0.04**
Saccular surface sagittal FLAIR	AUC=0.62, ***p** **=*** **0.04**	AUC=0.51, *p =* 0.93
Saccule/Utricle surface ratio sagittal FLAIR	AUC=0.56, *p =* 0.32	AUC=0.56, *p =* 0.32
Endolymphatic/total vestibule surface axial FLAIR	AUC= 0.52, *p =* 0.71	AUC= 0.54, *p =* 0.59
Saccular Height/Width ratio coronal FLAIR	AUC= 0.52, *p =* 0.72	AUC= 0.53, *p =* 0.59
Saccular Height/Width ratio coronal T2	AUC=0.501, *p =* 0.99	AUC=0.50, *p =* 0.99

EcoG (SP/AP > 0.33) was selected as an indicator of the Meniere disease. Performances are expressed by average area under the curve (AUC) and P-value (one-sample test, comparison to 0.5).

Separate ROC analysis of definite and probable unilateral MD showed that saccule and utricle surfaces on sagittal FLAIR views had a higher performance to discriminate affected ears in DMD than in PMD as judged by AUC (Table 9).

**Table 9:** Performance of MRI criteria by ROC analysis in definite and probable unilateral Meniere's disease (UMD).

**MRI Criteria**	**Definite UMD** **(*n =* 90 ears)**	**Probable UMD** **(*n =* 34 ears)**
Utricle surface sagittal FLAIR	0.71 ±0.060, ***p** **=*** **0.002**	0.52 ± 0.105, *p =* 0.81
Saccular surface sagittal FLAIR	0.80 ± 0.053, ***p** **<*** **0.0001**	0.55 ± 0.108, *p =* 0.59
Saccule/Utricle surface ratio sagittal FLAIR	0.83 ± 0.051, ***p** **<*** **0.0001**	0.51 ± 0.112, *p =* 0.90
Endolymphatic/total vestibule surface axial FLAIR	0.67 ± 0.063, ***p** **=*** **0.016**	0.53 ± 0.104, *p =* 0.706
Saccular Height/Width ratio coronal FLAIR	0.57 ± 0.067, *p =* 0.33	0.53 ± 0.106, *p =* 0.79
Saccular Height/Width ratio coronal T2	0.64 ± 0.062, ***p** **=*** **0.029**	0.62 ± 0.067, *p =* 0.08

Performances are expressed by average area under the curve (AUC) ± standard error and P-value (one-sample test, comparison to 0.5).

### 3.6. Logistic regression analysis

A model combining MRI criteria and audiovestibular data was explored to predict the clinically affected ear. Among all possible combinations between MRI criteria, PTA on audiometry, caloric weakness, vHIT, VEMP loss or asymmetry, ECoG (SP/AP > 0.33), the model which best predicted the affected ear based on clinical criteria was the combination of SURI (yes/no) and caloric weakness (yes/no). This model yielded the following characteristics: R^2^ = 0.22, Log likelihood = −66.2, R_SURI_ = 0.26, R_caloric_ = 0.15, and likelihood ratio: Chi-2 = 37.74, *p* < 0.0001. This combination improved the positive likelihood ratio of SURI. With this model, 44 out of 51 (86%) unaffected ears were predicted as normal, and 50 out of 75 affected ears (67%) were predicted as abnormal (specificity: 86%, and sensitivity: 67%).

### 3.7. Comparison of same-day MRI to delayed imaging

According to SURI criteria, ears with EH on same-day MRIs had greater hearing loss than those without hydrops, while in the delayed-MRI group, there was no difference in PTA between EH and no EH group (Table 5). In contrast, caloric and cVEMP loss appeared to be related to radiological EH both in the same-day MRI and the delayed-imaging groups if SURI or axial vestibular surface criteria on FLAIR views were considered (Tables 5, 10). Audiovestibular findings did not seem to be influenced by the imaging delay if other hydrops criteria were employed (Tables 5, 10). Interestingly, cVEMP loss appeared to be more frequent in patients no sign of EH according to height/width criteria on T2 sequences than in those with EH in the same-day MRI group (Table 5) underlining the possible dissociations between electrophysiology and MRI data.

**Table 10:** Relation between MRI hydrops criteria and delay between MRI and audiovestibular exams.

**MRI delay**	**1**−**10 days**			**11**−**60 days**			> **60 days**		
**SURI**	+	−	***p-v*** **alue**	+	−	***p-v*** **alue**	+	−	***p-v*** **alue**
PTA	49 ± 20.3 (2), 49[35−64]	57 ± 55.7 (4), 54[9−110]	1.0	42 ± 39.9 (5), 49[0−100]	51 ± 37.0 (9), 36[13−120]	0.5050	−	54 ± 47.9 (4), 53[10−100]	−
Caloric	1/2	2/4	1.0	3/3	2/9	**0.0180**	−	2/4	−
cVEMPs	1/2	0/2	0.2482	2/3	0/9	**0.0073**	−	0/4	−
ECoG	1/2	0/2	0.2482	2/5	6/9	0.3340	−	3/4	−
**FLAIR V.S**.	+	−	***p-v*** **alue**	+	−	***p-v*** **alue**	+	−	***p-v*** **alue**
PTA	35 (1)	41 ± 39.7 (10), 29[0−120]	0.7697	65 ± 23.4 (4), 56 [49−100]	41 ± 39.7 (10), 29 [0−120]	0.1039	15 (1)	66.7 ± 49.3 (3), 90[10−100]	0.6547
Caloric	1/1	2/5	0.2733	4/4	1/8	**0.0038**	0/1	2/3	0.2482
cVEMPs	0/1	1/3	0.5050	2/4	0/8	**0.0285**	0/1	0/3	−
ECoG	0/1	1/3	0.5050	2/4	6/10	0.7327	1/1	2/3	0.5050
**FLAIR H/W**	+	−	***p-v*** **alue**	+	−	***p-v*** **alue**	+	−	***p-v*** **alue**
PTA	43 ± 39.0 (5), 35[9−100]	110 (1)	0.1432	48 ± 36.8 (14), 43 [0−120]	−	−	54 ± 47.9 (4), 53 [10−100]	−	−
Caloric	3/5	0/1	0.2733	5/12	−	−	2/4	−	−
cVEMPs	1/4	−	−	0/4	−	−	−	−	−
ECoG	1/4	−	−	8/14	−	−	3/4	−	−
**T2 H/W**	+	−	***p-v*** **alue**	+	−	***p-v*** **alue**	+	−	***p-v*** **alue**
PTA	43 ± 34.6 (14), 45[8−110]	−	−	47 ± 34.3 (20), 38 [0−120]	55 (1)	0.6203	57 ± 43.7 (6), 59 [10−100]	−	−
Caloric	4/14	−	−	7/11	1/1	0.4602	2/2	−	−
cVEMPs	2/4	−	−	2/10	0/1	0.6572	0/4	−	−
ECoG	6/8	1/2	0.8489	9/15	−	−	3/4	−	−

Hydrops (+) or no hydrops (−) were defined on axial FLAIR-3D + gadolinium MRI views by saccule utricle ratio inversion (SURI), vestibular endolymphatic/total axial surface ratio (FLAIR V.S.), Saccular height and width criteria on coronal views (height > 1.6 mm or a height/width ratio < 1.14, FLAIR H/W), and on the same criteria on T2-weighed images (T2 H/W). For each criterion, audio vestibular test, fractions indicate the number of positive tests in favor of hydrops/total in each subgroup. Pure-tone average (PTA) is expressed as mean ± standard deviation (n), median, [min.-max.]. P-values are expressed for comparisons between hydrops (+) and no hydrops (−) by Mann-Whitney test for continuous variables and Chi-2 test for categorical parameters.

## 4. Discussion

In the last decade, many reports have demonstrated the utility of 3T-MRI in MD by visualizing the endolymphatic hydrops (22). This visualization can be pivotal in the diagnosis of clinically complex cases but also important for the follow-up or the evaluation of treatments (4, 22).

In this study, we showed that among diagnostic criteria of EH on MRI, SURI showed the best correspondence to the symptomatic side and audiovestibular findings. Saccule morphology on T2- weighted images did not seem coherent with clinical, audiovestibular and 3D-FLAIR findings. EH showed a better correspondence to hearing loss and vestibular weakness on same-day MRI than on delayed imaging at a different day.

### 4.1. Audiovestibular findings

It is noteworthy that this transversal study included all patients seen for the first time for a MD with a significant heterogeneity in terms of age, duration of the disease, severity, and ongoing treatment. This heterogeneity is typical in MD and reported in other publications (3, 4). At the time of inclusion, no therapeutic change was imposed, and the type of ongoing treatment was not set as an inclusion criterion because this type of restriction would have reduced the number of inclusions and the generalizability of the observations. Two patients (3% of the population) were treated with gentamicin. It is therefore important to note that the results of audiovestibular test in these patients might introduce a bias due to the potential vestibulotoxic and (to a lesser degree) cochleotoxic effect of the drug. These patients should perhaps not have been included in this study in the first place as their audiovestibular function may have been affected by the *in-situ* treatment.

ECoG results were bilaterally in most UMD patients. Similar results were found with comparable ratio SP/AP and area ratio of SP to AP between ispsilateral and contralateral ears in UMD patients (27). In contrast, another study comparing healthy and diseased ears in UMD patients found a difference in the SP response at 1 kHz, and the SP/AP ratio between the two ears (9). Also, several research teams describe a low sensitivity of the ECoG to confirm Meniere's disease, especially when using click (which is the case in our study) (9, 28). In our study, we find rather the opposite: as the contralateral ears display pathological results in the UMD group, the sensitivity of the ECoG is rather to high with a low specificity. Another study to investigate the sensitivity and specificity of ECoG to discriminate between healthy and “Meniere's” ears in UMD patients according to the use of clicks or tone burst would be interesting to develop.

Concerning UMD patients, cVEMP revealed relative reduction of amplitudes on the ipsi- or the contralateral side. It's important to precise that a relative reduction of cVEMP amplitudes on the contralateral side does not necessarily indicate a pathology on the contralateral side. In fact, it might also be due to a relative elevation of the cVEMP amplitude on the MD side, as has been observed before particularly in early stages of MD (29).

### 4.2. MRI findings

In contrast to other series (30), our semiquantitative analysis did not show significant hydrops in many cases (for example, VH grading *p* = 0.79 and CH grading *p* = 0.46, in UMD, *vs*. opposite ear, Table 4). However, quantitative analysis such as height and width criteria led to the diagnosis of hydrops in 92% of DMD and in 75% of PMD (Table 2). SURI criterion was also present in nearly 70% of ears with DMD. This discrepancy suggests that semiquantitative criteria (VH and CH grading) are probably less sensitive than quantitative measures and many abnormal ears were classed as grade 0 in the semiquantitative scale. To support this idea, another team enhanced the performances of the semiquantitative vestibular hydrops scale by adding a lower grade between 0 and 1 and proposed a four-grade classification (12).

In the first reports of this imaging technique, an intratympanic injection of gadolinium was employed to opacify only the perilymphatic space and to visualize the endolymphatic sector by contrast (10, 13–15). With this technique, the dilatation of the endolymphatic space can be seen in the vestibular and cochlear regions as black spots in a contrast-enhanced perilymphatic space (10, 13). Although reliable, this technique was progressively abandoned in favor of intravenous gadolinium (16, 18, 26, 31, 32). To reach the perilympahtic space through this route, the contrast agent requires several hours (16), and this delay often complicates the examination protocol in the routine practice. As an alternative, evaluation of the saccular anatomy on T2- weighted images has been proposed to avoid gadolinium (11). With this technique, the saccule could be clearly visualized on coronal slices, and its size and shape quantified. Hydrops was reportedly associated with an enlargement of this structure taking a more circular shape (11). By avoiding the gadolinium injection, this technique could be much easier to organize and would be better integrated in the routine practice. However, the analysis of our series was disappointing by showing no correlation between 3D-FLAIR and T2, revealing no relation between clinical or audiovestibular signs and T2-images, and showing a low discrimination of symptomatic ears on ROC analysis.

The fuzzy limits of the endolymphatic space on T2 sequences might have hampered the exact evaluation of the saccular size. Also, the fact that a small hydrops with no clinical signs may be present on the contralateral ear of a unilateral MD reduces the discrimination between affected and unaffected ears.

One of the most interesting findings of 3T-MRI is the presence of EH in asymptomatic ears of MD patients and even in normal subjects (17, 18). This finding corroborates histopathological studies showing endolymphatic enlargement in subjects with various diseases other than MD (e.g., otosclerosis, idiopathic progressive hearing loss), and even in those with no reported otological symptoms (21, 32). These observations suggest that the pathological mechanisms leading to EH are not fully understood (33, 34). EH seems to be merely an indicator of abnormalities in inner ear fluid homeostasis and it cannot solely explain the symptoms in MD (35). This further complexifies the relation between EH and audiovestibular findings in MD patients. Today, 3T-MRI data suggests that this phenomenon is far from anectodical, and this reduces considerably the specificity of EH in MD diagnosis (17, 18). The clinical and the pathophysiological significances of this phenomenon are not clear, and the crucial question in UMD is whether the presence of endolymphatic hydrops in the contralateral ear augurs poorly, indicating a BMD in the future. To our knowledge, there is no report focusing on this subject. The relatively low number of reported BMD (36) in comparison to the proportion of bilateral or contralateral EH on MRI is reassuring (18), but only a long-term follow-up of patients with UMD and bilateral EH will provide a direct answer (36).

### 4.3. Reversibility of MRI and audiovestibular results

Another interesting question is the reversibility of the endolymphatic space enlargement between two vertigo attacks. Intuitively, reversibility of the symptoms and the audiovestibular signs should be accompanied by a possible regression of the hydrops on imaging. But the kinetics of the electrophysiological alterations and the morphological signs are probably different (37, 38) and the observed lag between a normalized function and a decrease of endolymphatic pressure can explain the absence of correlation between audiovestibular findings and MRI observations (38). Another factor which can disturb such correlations is the extent of irreversible cochleovestibular damage caused by gentamycine, age, long-term hydrops, or comorbidity factors which do not vary with the hydrops. These elements indicate that, MRI should be regarded as one additional diagnostic tool, and not a gold standard for the diagnosis and the treatment of MD. This tool can be useful to support or confirm the diagnosis in clinically atypical cases or in the early stages of the disease with only cochlear or vestibular symptoms (3, 4). It would be interesting to discuss its integration to the international MD diagnostic criteria: MRI signs of EH in a probable MD could lead to reclassify the case into definite MD. In fact, those patients are less likely to present with EH on MRI, as has been shown in the present study (lower rate of EH in PMD than DMD) and previous studies (39).

### 4.4. Limits of MRI outcomes

A common limit in all MRI-based morphological studies of EH is that the threshold at which the diagnosis is established is crucial for both sensitivity and specificity of the MRI. These thresholds are based on relatively small number of cases (n< 70) (10, 11, 17). Moreover, sensitivity and specificity of these tests are based on the clinical diagnosis of MD which is not constantly associated to histopathological deteriorations during EH (20). Alternatively, the use of electrophysiological indicators (e.g., SP/AP on ECoG) for this purpose has been disappointing (40, 41).

One of the limitations of the surface methods to evaluate hydrops probably comes from the fact that the endolymphatic spaces show a significant interindividual variation even in healthy ears (42). Indeed, on a sagittal medial plane, the saccule could be detected in only 15 among 22 examined healthy ears on 3D-FLAIR MRI sequences after gadolinium. The endolymphatic space had also a variable shape in the axial plane, often Y-shaped (77%) but globular in other cases (23%).

Another limit is that MRI approaches its resolution limits in morphological studies of small structures such as the inner ear. Reliability of linear measurements between 1 and 2 mm on MRI is low since they are close to the slice thickness (43, 44). This is probably one of the reasons why criteria such as SURI and semi quantitative classifications of vestibular and cochlear hydrops seem more coherent to clinical data than diameter and surface measurements. Low resolution might also explain false negative cases. Indeed, the dilatation of the cochlear duct, which is difficult to visualize, is a constant histopathological observation in MD while saccular and utricular enlargements are noted on MRI in only 80 and 55% of temporal bones respectively (45). Focal dilatations of the cochlear duct are also frequently observed in MD and other diseases (33) and are probably underestimated on 3T-MRI (18). Our study also suffers from several limitations proper to its design. The heterogeneity of the population in terms of disease duration, age and medication can introduce discrepancies in terms of relation between audiovestibular findings and MRI. Indeed, recent MD will present with smaller hearing loss and little or no caloric weakness but possibly significant EH on MRI while MD progressing for several years may show more sever audiovestibular deficit with similar or smaller EH on MRI. Even though every precaution was taken during the examination and the classification of the MRI images, interpretation of the images by 2 independent radiologists would have added to the precision of the data.

MRI resolution is directly dependent on the signal/noise ratio and this parameter could be physically improved by increasing the magnetic field force (i.e., 7T-MRI used in research field) or acquisition time (46, 47). A specific antenna close to the target can also enhance SNR (48). In future, signal processing algorithms will also better detect signal in noise and improve image quality and resolution (49).

In conclusion, among several EH criteria, SURI had the strongest relation to audiovestibular and clinical MD manifestations. There was no significant relation between SURI and other MRI criteria for EH even in ears with definite MD suggesting that they evaluate different aspects of the EH and a composite criterion might be more suitable to evaluate the EH. A short delay between MRI and audio vestibular testing seemed to improve coherence between these tests and EH diagnosis on MRI.

## Data availability statement

The original contributions presented in the study are included in the article/supplementary material, further inquiries can be directed to the corresponding author.

## Ethics statement

The studies involving human participants were reviewed and approved by CPP Est I, number: 20016-A00875-46. The patients/participants provided their written informed consent to participate in this study.

## Author contributions

SD: conception or design of the work, acquisition and analysis of data for the work, drafting the work, final approval of the version to be published, and agreed to be accountable for all aspects of the work in ensuring that questions related to the accuracy or integrity of any part of the work are appropriately investigated and resolved. CG: conception or design of the work, analysis of data for the work, revising the work critically for important intellectual content, final approval of the version to be published, and agreed to be accountable for all aspects of the work in ensuring that questions related to the accuracy or integrity of any part of the work are appropriately investigated and resolved. ED and J-LB: acquisition and interpretation of data for the work, revising the work critically for important intellectual content, final approval of the version to be published, and agreement to be accountable for all aspects of the work in ensuring that questions related to the accuracy or integrity of any part of the work are appropriately investigated and resolved. MT: conception or design of the work, revising the work critically for important intellectual content, final approval of the version to be published, and agreement to be accountable for all aspects of the work in ensuring that questions related to the accuracy or integrity of any part of the work are appropriately investigated and resolved. AB-G: conception or design of the work, acquisition, analysis, and interpretation of data for the work, drafting the work, final approval of the version to be published, and agreement to be accountable for all aspects of the work in ensuring that questions related to the accuracy or integrity of any part of the work are appropriately investigated and resolved. All authors contributed to the article and approved the submitted version.
